# Gastroesophageal reflux in Bronchiectasis and the effect of anti-reflux treatment

**DOI:** 10.1186/1471-2466-13-34

**Published:** 2013-06-03

**Authors:** Zhi-Wei Hu, Zhong-Gao Wang, Yu Zhang, Ji-Min Wu, Jian-Jun Liu, Fang-Fang Lu, Guang-Chang Zhu, Wei-Tao Liang

**Affiliations:** 1 Xuanwu Hospital of Capital Medical University, No. 45 Changchun Road, Xicheng District, Beijing 100053, China; 2 Center for GER, the Second Artillery General Hospital, Beijing Normal University, No. 16 Xinwai Road, Xicheng District, Beijing 100088, China

**Keywords:** Gastroesophageal reflux, Bronchiectasis, Asthma, Stretta radiofrequency, Laparoscopic fundoplication

## Abstract

**Background:**

Bronchiectasis is a progressive and fatal disease despite the available treatment regimens. Gastroesophageal reflux (GER) may play an important role in the progression of bronchiectasis. However, active anti-reflux intervention such as Stretta radiofrequency (SRF) and/or laparoscopic fundoplication (LF) have rarely been used to treat Bronchiectasis.

**Case Presentation:**

Seven patients’ clinical outcomes for treating GER-related deteriorated bronchiectasis were retrospective reviewed. All patients were treated by SRF and/or LF, and had follow-up periods ranging from one to five years. Typical GER symptoms, respiratory symptoms, medication consumption and general health status were assessed during the follow-ups. At the latest follow-up all patients were alive. The typical GER symptoms disappeared in five people and were significantly improved in the other two. Two had complete remissions of both respiratory symptoms and bronchiectasis exacerbations; four had significantly improved respiratory symptoms to mild/moderate degrees as well as reduced or zero bronchiectasis exacerbations, which allowed them to resume the physical and social functions; one’s respiratory symptoms and bronchiectasis exacerbations were not much improved, yet she was in stable condition and satisfied with the results.

**Conclusions:**

Potentially, GER plays an important role in some patients with bronchiectasis, and active anti-reflux treatments can be beneficial. Future clinical studies are suggested to clarify GER’s role in bronchiectasis and to further determine whether anti-reflux interventions for GER can improve the outcomes of patients with bronchiectasis.

## Background

Bronchiectasis is defined as an irreversible dilatation of the bronchial tree, representing the end stage of a number of different pulmonary pathology [1]. Bronchiectasis is usually a complication of previous lower respiratory infection, and causes chronic cough and copious production of sputum, which is often purulent. The prevalence of bronchiectasis in the United States ranges from 4.2 per 100,000 persons aged 18–34 years to 271.8 per 100,000 persons aged 75 years or older [2]. It occurs more in elderly than in younger adults and more in women than in men. Bronchiectasis causes severe pulmonary infections and deteriorates lung function. It can also be associated with cystic fibrosis and other congenital pulmonary disorders, foreign body inhalation, and other causes of lung damage [3]. Frequent exacerbations are often associated with infection and symptoms of increased dyspnea, wheeze, and sputum production [4].

As the disease progresses, patients’ physical activities become so increasingly limited that they fail to thrive, thus ultimately suffer from social deprivation, intrinsic depression, respiratory failure, chronic morbidity, and even the possibility of premature mortality [5,6]. The outcome of bronchiectasis has improved during the past decades, but it still remains as an important cause of death. The bronchiectasis related mortality seems to be up to 13% over a 5-year follow-up period [7]. The treatment of bronchiectasis is multimodality which includes therapy with antibiotics, anti-inflammatory agents and airway clearance, and requires pulmonary surgery and even lung transplantation in a few selected patients [8]. GER which may be a possible etiology and risk factors associated with bronchiectasis have not been well addressed. Among patients with late stages of lung diseases, the opportunity of treating GER is only shown in a limited studies for chronic obstructive pulmonary disease and idiopathic pulmonary fibrosis, and in even fewer studies for bronchiectasis [9]. For the past 6 years we have focused on treating GER related respiratory symptoms [10,11], and our fundamental viewpoint is that GER is an important risk factor for recurrent microaspiration, which may cause airway irritation, lung injury and respiratory tree remodeling [12]. Hereby, in order to explore the importance of GER’s role in the progression of bronchiectasis, we retrospectively reviewed a series of selected bronchiectasis patients whose symptoms and physical functions were either stabilized or improved after the anti-reflux interventions.

### Patients and methods

In this series, patients were selected from Center for GER, the Second Artillery General Hospital of Beijing Normal University, China, from 2008 to 2012. The patients’ typical GER symptoms, respiratory symptoms, medication consumption and general health status were assessed before and after anti-reflux intervention. This study was carried out with the approval of the Ethics Committee of Second Artillery General Hospital.

#### Inclusion criteria

**Figure 1:**
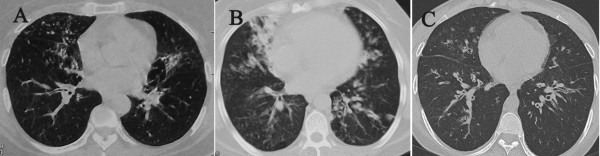
Chest CT of case 4 (A), 6 (B) and 7 (C), which demonstrates diffuse dilated and thickened bronchi accompanied with lung fibrosis.

1. Bronchiectasis was diagnosed according to the patient’s longstanding respiratory symptoms, chest film and computed tomography (CT) (Figure 1); 2. The presence of GER were determined by various testing methods; 3. Patients were only partially responsive to medication therapy for Bronchiectasis; 4. Patients received SRF and/or LF.

#### GER assessment and treatment

All the following tests were conducted to confirm the presence of GER: 1. Ambulatory 24-h esophageal pH probe monitoring, and a DeMeester score (DMS) > 14.72 was considered positive for increased acid GER [13]; 2. Endoscopic evaluations for esophagitis and hiatus hernia (HH); 3. High Resolution Manometry (HRM) for elucidating the function of upper esophageal sphincter (UES) and lower esophageal sphincter (LES).

The methods for how to carry out the SRF and the LF were described in our previous studies [14,15].

## Case presentation

One male and six female patients were included. Their mean age was 49 years (ranging from 22 to 70 years). The patients’ baseline pulmonary evaluation results are summarized in Table 1. The results for patients’ GER baseline evaluations, treatments and follow-ups are shown in Table 2.

**Table 1:** The baseline of patients’ lung function test at admission

**Variables**	**Case 1**	**Case 2**	**Case 3**	**Case 4**	**Case 5**	**Case 6**	**Case 7**
FVC, L (% predicted)	3.17 (75)	1.37 (40)	2.48 (96)	2.85 (100)	1.82 (50)	1.86 (65)	3.13 (81)
FEV1, L (% predicted)	0.82 (31)	0.93 (32)	1.72 (84)	1.68 (73)	1.04 (37)	0.68 (29)	1.29 (40)
FEF, L/sec (% predicted)	2.54 (42)	3.29 (53)	4.48 (84)	3.72 (66)	3.17 (42)	2.21 (39)	2.87 (42)
FEV1/FVC, %	34	68	69	59	57	37	41

*FVC*, Forced vital capacity; *FEV1*, Forced expiratory volume in one second; *FEF*, Forced expiratory flow.

**Table 2:** Patients’ GER evaluation at admission, treatment and follow-up

**Case**	**Treatment**	**Follow-up**	**GER symptom**	**Endoscopy**	**24-h pH**	**HRM**
Case 1	LF	5-yr.	Evident and progressive daily regurgitation, heartburn and chest pain for 10 years.	LA-B	DMS: 3.04	MUESP: 53.1
						MLESP: 14.0
						LHPZ: 3.3
Reexamine			Asymptomatic	negative	DMS: 1.13	—
Case 2	LF	4-yr.	Occasional regurgitation heartburn and vomiting in cough exacerbations for 5 years.	LA-A; HH	DMS: 136.06	MUESP:59.1
						MLESP: 16.2
						LHPZ: 4.0
Reexamine			Asymptomatic	negative	DMS: 1.40	—
Case 3	LF	4-yr.	Daily regurgitation, heartburn and chest pain since childhood, worsened in resent 2 years.	HH	DMS: 3.89	MUESP: 23.7
						MLESP: 7.4
						LHPZ: 1.5
Reexamine			Asymptomatic	negative	DMS: 2.24	—
Case 4	SRP	2-yr.	Weekly or daily regurgitation and heartburn for 4 years.	negative	DMS:1.93	MUESP: 40.3
						MLESP: 24.0
						LHPZ: 4.3
Reexamine			Asymptomatic	negative	DMS:1.30	—
Case 5	SRP	.2-yr.	Weekly regurgitation, heartburn and chest pain for 20 years	LA-A	DMS: 17.90	MUESP: 22.6
						MLESP: 23.1
						LHPZ: 3.4
Reexamine			Become occasional with daily PPI	LA-A	DMS: 8.45	—
Case 6	LF + SRP	.1-yr.	Occasional regurgitation, belching and heartburn for 10 years.	LA-A; HH	DMS: 19.87	MUESP: 35.5
						MLESP: 11.8
						LHPZ: 3.0
Reexamine			Asymptomatic	negative	DMS: 1.10	—
Case 7	LF	.1-yr.	Occasional heartburn for 10 years.	LA-A	DMS: 60.0	MUESP:63.4
						MLESP: 21.0
						LHPZ: 4.1
Reexamine			Asymptomatic	LA-A	DMS: 2.30	—

*HRM*, High-resolution manometry; *DMS*, DeMeester score; *LA*, Los Angeles classification (LA-A indicates one or more mucosal breaks of ≤5 mm in length, LA-A one or more mucosal breaks of >5 mm); *HRM,* High-resolution manometry; *MUESP*, Mean upper esophageal sphincter pressure (Normal range: 34–104 mmHg); *MLESP,* Mean lower esophageal sphincter pressure (Normal range: 13–43 mmHg); *LHPZ*, Length of high pressure zone (Normal range: 2.7-4.8 cm), — Not reexamined.

Case 1: The patient was a 48-year-old woman, whose asthma was diagnosed at 8 months old. Twenty one years ago she was diagnosed as bronchiectasis based on a chest CT scan, and since then she had to be hospitalized several times every year for severe exacerbations. Her symptoms were characterized as increased wheezing, chest tightness, and productive cough with massive brownish sputum which mainly occurred in the early morning. Ten years ago she started to have symptomatic GER and deteriorated exertional dyspnea. In August 2008 the patient received LF. Five years after the intervention, the patient had no GER symptoms, her episodic wheezing and chest tightness were reduced about 30% without hospitalization, and her respiratory drug consumption was cut by more than half. Although there were fewer improvements in her cough and sputum productions, the brownish sputum became occasional.

Case 2: Bronchiectasis was diagnosed in this 30-year-old woman based on typical clinical features and CT scan 7 years ago. Since she was born, the patient had intermittent symptoms of severe cough, sputum production as well as dyspnea mainly at night. Since then, she had to be repeatedly hospitalized due to exacerbations every year. She also developed mild GER symptoms, accompanied with daily respiratory symptoms and severe dyspnea on exertion and fatigue in the later 5 years. In September 2009 the patient underwent laparoscopic repair of HH with LF. She became asymptomatic and had resumed normal life without medication based on her 4-year follow-up. Although the patient reported mild cough and fatigue during her pregnancy in the later 6 months, she was still satisfied with our therapy.

Case 3: The patient was 70 years old. Her bronchiectasis was diagnosed by a chest film 42 years ago after her first episode of hemoptysis during sleep and was later confirmed by a CT scan 15 years ago. There was no severe cough or sputum production in her past history. Her bronchiectasis was relatively stable and was mainly represented as hemoptysis and slow progression of fatigue. However, she complained much more about the severe GER symptoms which was developed since her childhood 60 years ago and worsened with reduced response to anti-reflux medication. Four years ago, a HH was confirmed by a barium swallow study. After evaluation we performed a laparoscopic HH repair with LF on the patient in October 2009. Without medication both the GER symptoms and hemoptysis disappeared, and the patient felt stable and more energetic at the 4-year follow-up.

Case 4: This was a 59-year-old woman who was diagnosed with asthma 20 years ago, and with bronchiectasis based on CT scans 10 years ago. Her respiratory symptoms had been accompanied with persistent GER symptoms for 20 years and had worsened with exertional dyspnea in the later 2 years. The respiratory symptoms represented as episodic throat tightness, wheezing, short of breath and productive cough. During the last 2 years, repeated emergency treatments were required due to deteriorated exacerbations. In April 2011, the patient received radiofrequency energy delivery to the LES, The SRF procedure. At the 2-year follow-up, she was on the daily dose of 20 mg Omeprazole. Her episodic throat tightness and wheezing disappeared, and short of breath and productive cough ameliorated more than half. Although not fully recovered, she was satisfied with her stable condition without hospitalization.

Case 5: The patient was a 59-year-old man who was diagnosed with asthma 30 years ago and with bronchiectasis based on chest film and CT scan 10 years ago. His symptoms were characterized by increased wheezing, chest tightness, productive cough which mainly occurred at night and early morning. He had numerous emergency visits due to severe exacerbations, in several of which he had throat tightness, inability to breath, intense stridor and syncope. The symptoms significantly worsened in the later 3 years with exertional dyspnea and incapable of less than 100 meter walk. For 4 years he also experienced symptomatic GER. In November 2011, the patient received SRF treatment. At the 2-year follow-up after the intervention, the patient had no GER symptoms and only mild productive cough without exacerbation and medication. His normal daily activity was resumed and was capable of housework and nonstop walking for two kilometers.

Case 6: The patient was 58 years old and was diagnosed with asthma 25 years ago and with bronchiectasis based on CT scan 4 years ago. The respiratory symptoms had lasted for 30 years and had worsened with disabling exertional dyspnea accompanied with GER symptoms for the later 10 years. Her symptoms presented as episodic throat tightness, wheezing, short of breath and productive cough. During the later 10 years she had daily symptoms and numerous emergency treatments for exacerbations. In September 2011 she underwent laparoscopic repair of the HH with LF. Afterwards, the patient’s GER symptoms became occasional, the episodic throat tightness and wheezing disappeared, and exertional dyspnea was markedly improved, yet her cough remained unchanged and had become bothersome for 6 months until she had an exacerbations and temperature reached 39.0°C. The patient was readmitted to our hospital. In March 2012, per her insistent request we performed SRF therapy when she was stabilized after a 14-day course of antibiotics. To our surprise, her cough was cleared and exertional dyspnea was further improved after SRF therapy. She resumed relatively normal daily activity as a housewife and was stable without medication afterwards.

Case 7: This was a 22-year-old junior college student, whose asthma was diagnosed since she was born and bronchiectasis was diagnosed based on a chest CT at admission. Her symptoms were characterized by heavy wheezing, chest tightness, productive cough of massive white foamy sputum which mainly occurred at night. For 15 years, she had symptomatic GER and worsening exertional dyspneas. She had received 4 emergency treatments for exacerbations, and in one of them she lost her consciousness. In May 2012, after we gave her a LF, her episodic productive cough and wheezing disappeared without medication, the exertional dyspnea was markedly improved, and she obtained a normalized college life.

## Discussion

The clinical course of bronchiectasis is generally punctuated by infectious exacerbations, and the clinical presentation of bronchiectasis may be complicated by the coexistence of other medical conditions or comorbidities, including GER [16,17]. The role of GER in the pathogenesis of miscellaneous respiratory symptoms and diseases has been discussed for decades and it is common understanding on its close association in asthma and cough. There is a high prevalence of reflux in asthmatics and chronic cough, which may be induced through different mechanisms including microaspiration and both local and central reflexes [18]. Symptomatic and clinically silent reflux has been detected in bronchiectasis, with the prevalence from 26% to 75%, and aspiration of gastric contents in the respiratory tree is not rare in patients with GER [19,20]. In a group of patients with advanced lung disease including bronchiectasis manometry showed that 57% of patients had LES hypotonia and 14% had UES hypotonia [21]. Similar results were found in the seven patients of our study. Four patients had evident GER symptoms and the other three only had occasional GER symptoms. Four patients had abnormal acid reflux detected by 24 hour esophageal pH monitoring and five patients had esophagitis. In addition, two patients were detected with LES hypotonia and two with UES hypotonia (Table 2). GER may have influence on respiratory symptoms or bronchiectasis with similar mechanisms, reflex bronchoconstriction, and pulmonary microaspiration [9,22]. The frequency and duration of episodes, as well as the volume, composition, and destination of GER are all factors in determining its significance [12]. With aspiration into the tracheobronchial tree, GER is hypothesized to present as insidious-onset bronchiectasis, which induces erosion via reflux contents thus triggering chronic airway inflammation and remodeling [23].

Increased GER is principally caused by gastroesophageal junction incompetence and may arise from LES hypotension, including transient relaxations, HH, and esophageal dysmotility [24]. Current GER therapy focuses on modifying risk factors, inhibiting the production of gastric acid, enhancing esophageal and gastric motility, and modifying gastroesophageal junction. In GER treatments, both proton pump inhibitor (PPI) and LF are highly successful in relieving esophagus symptoms [25]. However, recent meta-analysis found no significant difference between placebo and PPI in resolving GER related cough and asthma [26]. The possible reason could be that anti-acid treatment provides fast symptom relief on GER, but does not sufficiently prevent aspiration. Moreover, the relapses could be as high as 70% within 6 months after the medication is stopped [27]. As presented in this case series, SRF or LF restores anatomical anti-reflux barriers and reduces GER symptoms and possibly prevents the related aspiration, thus can serve as a better therapeutic means for bronchiectasis. The anti-reflux surgeries reported encouraging results in asthmatic patients selected on the basis of esophageal pH monitoring [28], and are well demonstrated in our previous LF and SRF studies [14,15,29-31]. Yet, the effects are unknown on modifying risk factors and acid inhibit therapy in bronchiectasis patients. LF has been performed for GER in end-stage lung diseases mainly including idiopathic pulmonary fibrosis, cystic fibrosis and COPD before or after lung transplantation, and is beneficial for lung functions, allograft and quality of life in the selected patients [32-35]. A report by Davis et al. showed that within a group of patents with lung transplantations, fundoplication in two patients with advanced bronchiectasis had resulted in lung function improvements and reduction in oxygen requirements [36]. In our study, LF was carried out in four patients, SRF was performed in two patients, and only one patient received both SRF and LF. The patients selected in our study were not in end-stage bronchiectasis requiring lung transplantation. After anti-reflux interventions, one patient was under daily dose of 20 mg Omeprazole for GER and six patients did not need PPI, while acid reflux was normalized in all the patients. Respiratory symptoms, physical and social functions were significantly improved in six patients and partially improved in one. Based on our experience, active anti-reflux intervention such as LF or SRF could be considered in the setting of GER related respiratory symptoms or diseases in various stages including the end-stage, as to achieve more precise, long-lasting and stable therapeutic effects. Especially, when HH is involved, laparoscopic surgery could be considered as the most effective means for GER.

Specific pathophysiological features which are characteristic of respiratory diseases such as chronic cough and asthma may accompany with bronchiectasis, to make it a more complicated disease. The prevalence of bronchiectasis among asthma patients was 1.8% and asthma in bronchiectasis patients was 19.0% [37]. Asthma patients who had coexisting bronchiectasis had significantly more hospitalizations due to severe asthma exacerbations and presence of chronic respiratory failures [38]. As found in our study, five of the seven patients had also been repeatedly diagnosed as asthma in other hospitals and required frequent hospitalizations. One hypothesis to explain the prevalence of bronchiectasis in asthma patients is that the hyperresponsive bronchi are destroyed by inflammatory process or recurrent infection. However, when GER is involved, both asthma and bronchiectasis may be the same consequence of GER, and in this term, GER, asthma and bronchiectasis can be considered as the same entity [10]. The longstanding respiratory diseases related to GER had negative impacts on lung functions of the seven patients. As shown in Table 1, all patients’ lung functions had declined to the COPD level (FEV1/FVC < 70%), with four of them in severe stages (FEV1 30%–49%) and one in very severe stage (FEV1 < 30%) (Table 1). Thus early evaluation and effective control of bronchiectasis related GER may result in more benefits for the bronchiectasis patients. As none of the patients reexamined spirometry in this series, whether anti-reflux intervention improves the patient’s pulmonary function is indicated in further studies.

**Figure 2:**
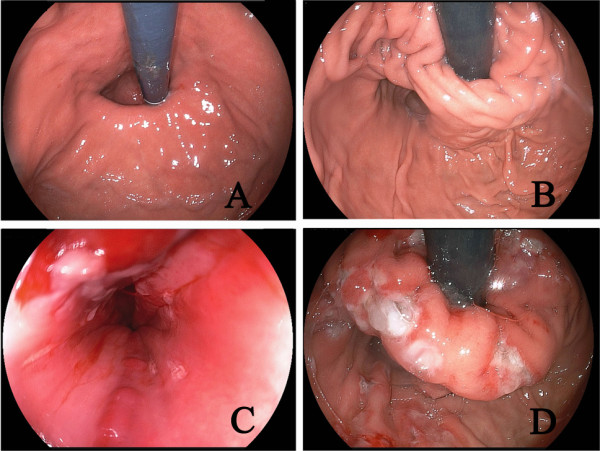
**In case 6, a sliding hernia was identified under endoscopy (A), which was considered as the cause of the patient’s GER and then the asthmatic symptoms.** This anatomical defect was then corrected by laparoscopic repair of the HH with LF (**B**). Although her respiratory symptoms was significantly relieved, the remaining cough was still evident, an additional anti-reflux SRF was conducted (**C** &**D**), which finally cleared her cough.

**Figure 3:**
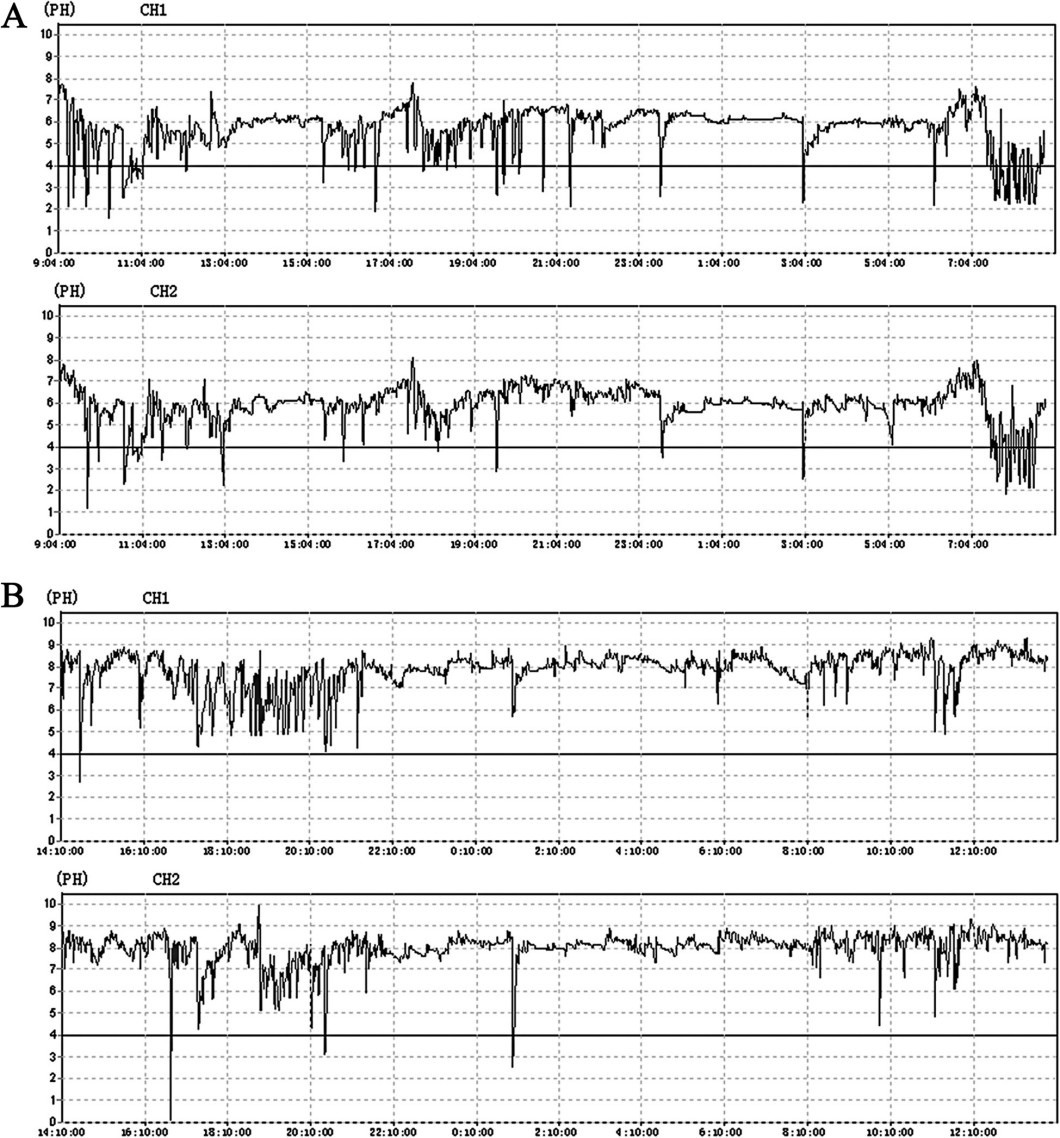
**24-h Dual-channel esophageal pH monitoring graphs shows the pH monitor line featuring the pH fluctuation in esophagus, <pH 4.0 indicating of acid reflux. A**: In case 7, the distal and proximal esophagus (CH1 and CH2) had synchronic pathologic acid reflux (DeMeester score: 60.0). **B**: In case 1, the pH line rarely declined below pH 4.0 (DeMeester score: 3.04). However her basal esophagus pH was >7, if a threshold of < pH 5 or < pH 6 (weak acid) is applied the patient should be considered to have abnormal synchronic distal and proximal reflux [42].

**Figure 4:**
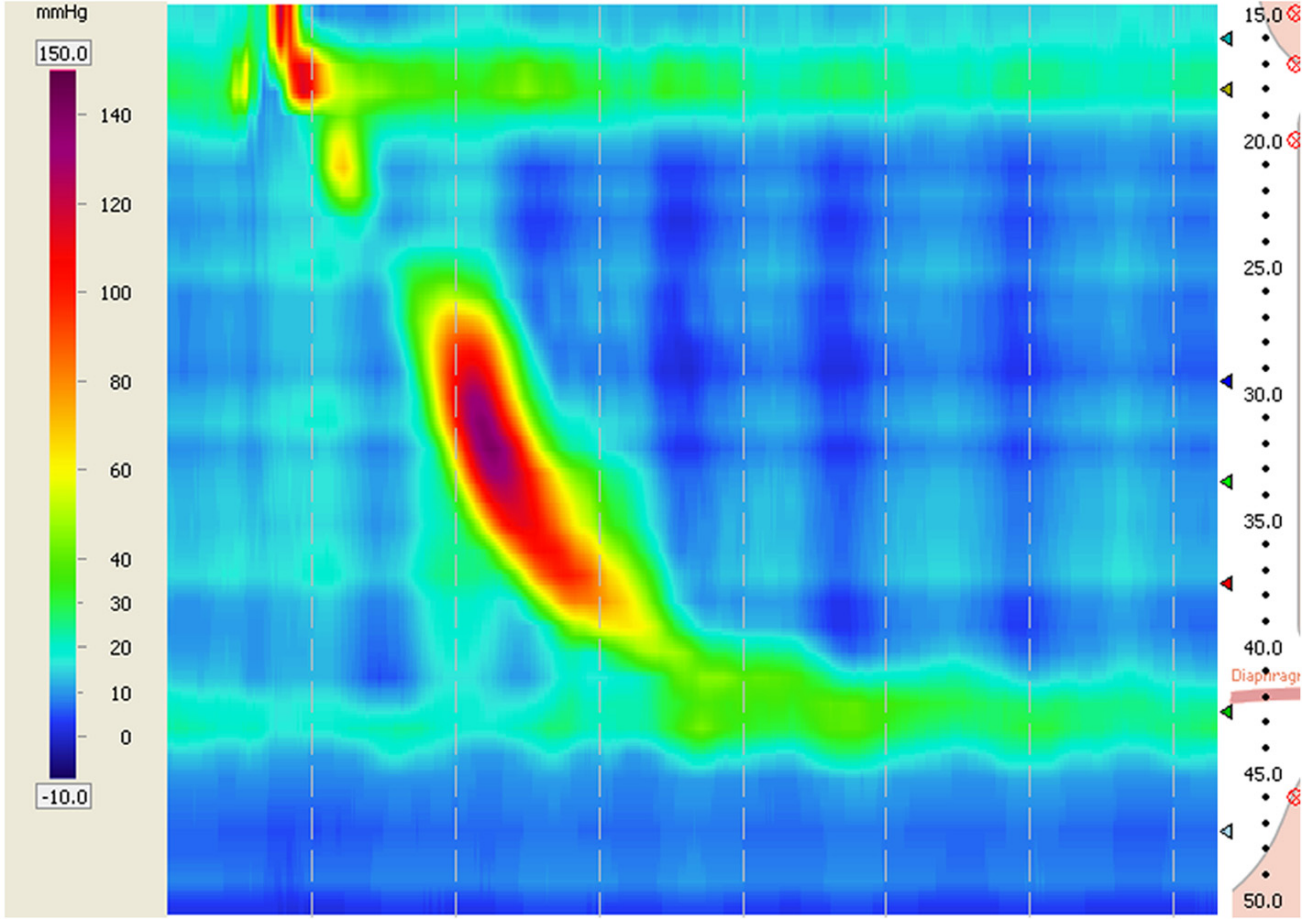
**High-resolution color contour of HRM.** In case 5, the body and LES of the esophagus function normally. However the UES resting pressure is in hypotension. The UES and LES are allies of the anti-reflux barrier either dysfunction of them can lead to trans-UES reflux which may cause microaspiration.

GER can be assessed in a number of ways. Endoscopy provides evidence of esophagitis and HH [39], and the presence of HH is an important indication for laparoscopic treatment in our practice (Figure 2). 24-h Dual-channel esophageal pH monitoring, the standard test for GER, may demonstrate abnormal esophageal acid exposure in the absence of esophageal damage (i.e. non-erosive disease), and demonstrates the pH fluctuation at the distal and proximal esophagus (Figure 3). Yet the development of esophageal impedance monitoring now allows the assessment of all reflux events (regardless of degrees of acidity) and further classification of reflux by the proximal extension, e.g. to the upper esophagus or even pharynx [40]. In addition, HRM demonstrates esophageal dyskinesia or hypotonia of the UES and LES (Figure 4), thus it elucidates the possible esophagus dysfunction involved in GER [41]. In our opinion, for patients with active bronchiectasis and/or asthma, a complete GER evaluation should be recommended, especially when they have evident GER symptoms, which may have important diagnostic and therapeutic importance for the patients.

## Conclusion

The limitations in the retrospective observations made in this case series are inevitable. However, given the lack of effective treatments for bronchiectasis and the encouraging results in these patients whose conditions may have otherwise deteriorated during the follow-up years, this study could be informative and provocative. A complete evaluation on bronchiectasis patients for treatments of the underlying causes such as GER may improve symptoms and prevent disease from progression. Further larger studies are needed for the following: (1) Determining the clinical significance of aspiration in bronchiectasis patients and the responses to anti-reflux treatments; (2) Finding out better diagnostic tests to predict which patients can have better outcomes if treated by anti-reflux interventions; (3) To understand how much of the bad status of bronchiectasis could be reversed.

## Consent

Written informed consents were obtained from the patients for publication of this case report and for any associated images. A copy of the written consents is available for review by the Editor-in-Chief of this journal.

## Abbreviations

GER: Gastroesophageal reflux; SRF: Stretta frequency; LF: Laparoscopic fundoplication; CT: Computed tomography; HRM: High-resolution manometry; DMS: DeMeester score; HH: Hiatal hernia; LES: Lower esophageal sphincter; PPI: Proton pump inhibitors; FVC: Forced vital capacity; FEV1: Forced expiratory volume in one second; FEF: Forced expiratory flow; LA: Los Angeles classification; MUESP: Mean upper esophageal sphincter pressure; MLESP: Mean lower esophageal sphincter pressure; LHPZ: Length of high pressure zone.

## Competing interests

The authors declare that they have no competing interests.

## Authors’ contributions

ZWH studied and analyzed the seven cases, conducted literature reviews, and drafted the manuscript. ZGW designed the study and helped to draft the manuscript. JMW, JJL, YZ, GCZ and WTL carried out the study, collected the data and helped to draft the manuscript. All authors read and approved the final manuscript.

## Authors’ information

ZW Hu, Y Zhang, FF Lu, GC Zhu and WT Liang the resident in training program in Center for GER of the Second Artillery General Hospital and Xuanwu hospital.

ZG Wang ( He himself was a so called severe asthma patient with severe and intolerable respiratory symptoms requiring medication all the time for years, after GER was confirmed and fundoplication performed, asthma disappeared completely without applying any medication afterwards [11]. Then he decided to devote himself to rescue those patients who suffered the same as him, therefore a GER Center established since 2006), the pioneer of GER related airaway diseases research and practice in China, the founder of Center for GER of the Second Artillery General Hospital, Professor and Director of Vascular Institute of Xuanwu Hospital of Capital Medical University Capital, Life Long President of Chinese Vascular Society, Vice President of International Society of Vascular Surgery.

JM Wu and JJ Liu, the director and chief physician of Center for GER of the Second Artillery General Hospital.

## Pre-publication history

The pre-publication history for this paper can be accessed here:

http://www.biomedcentral.com/1471-2466/13/34/prepub
